# Delivering Team-Based Primary Care for the Management of Chronic Low Back Pain: An Interpretive Description Qualitative Study of Healthcare Provider Perspectives

**DOI:** 10.1080/24740527.2023.2226719

**Published:** 2023-09-08

**Authors:** Kyle Vader, Catherine Donnelly, Therese Lane, Gillian Newman, Dean A. Tripp, Jordan Miller

**Affiliations:** a School of Rehabilitation Therapy, Queen’s University, Kingston, Ontario, Canada; b Chronic Pain Network, McMaster University, Hamilton, Ontario, Canada; c Canadian Arthritis Patient Alliance, Toronto, Ontario, Canada; d Patient Engagement Research Ambassadors, Institute of Musculoskeletal Health and Arthritis, Canadian Institutes of Health Research, Toronto, Ontario, Canada; e Curvy Girls Scoliosis, Toronto, Ontario, Canada; f Department of Psychology, Queen’s University, Kingston, Ontario, Canada; g Department of Anesthesiology, Queen’s University, Kingston, Ontario, Canada; h Department of Urology, Queen’s University, Kingston, Ontario, Canada

**Keywords:** Low back pain, team-based care, primary care, focus groups, qualitative research

## Abstract

**Background:**

Chronic low back pain (LBP) is a prevalent and disabling health issue. Team-based models of primary care are ideally positioned to provide comprehensive care for patients with chronic LBP. A better understanding of primary care team perspectives can inform future efforts to improve how team-based care is provided for patients with chronic LBP in this practice setting.

**Aims:**

The aim of this study was to understand health care providers’ experiences, perceived barriers and facilitators, and recommendations when providing team-based primary care for the management of chronic LBP.

**Methods:**

We conducted an interpretive description qualitative study based on focus group discussions with health care providers from team-based primary care settings in Ontario, Canada. Data were analyzed using thematic analysis.

**Results:**

We conducted five focus groups with five different primary care teams, including a total of 31 health care providers. We constructed four themes (each with subthemes) related to experiences, perceived barriers and facilitators, and recommendations to providing team-based primary care for the management of chronic LBP, including (1) care pathways and models of service delivery, (2) team processes and organization, (3) team culture and environment, and (4) patient needs and readiness.

**Conclusions:**

Primary care teams are implementing diverse care pathways and models of service delivery for the management of patients with chronic LBP, which can be influenced by patient, team, and organizational factors. Results have potential implications for future research and practice innovations to improve how team-based primary care is delivered for patients with chronic LBP.

## RÉSUMÉ

**Contexte:** La lombalgie chronique est un probléme de santé répandu et invalidant. Les modéles de soins primaires en équipe sont particuliérement bien placés pour dispenser des soins complets aux patients souffrant de lombalgie chronique. Une meilleure compréhension des perspectives de l’équipe de soins primaires peut éclairer les efforts futurs visant à améliorer la façon dont les soins en équipe sont dispensés aux patients atteints de lombalgie chronique dans ce cadre de pratique.

**Objectifs:** L’objectif de cette étude était de comprendre les expériences des prestataires de soins de santé, les obstacles et les facilitateurs perçus, ainsi que les recommandations pour la prestation de soins primaires en équipe dans le cadre de la prise en charge de la lombalgie chronique.

**Méthodes:** Nous avons mené une étude qualitative de description interprétative basée sur des discussions de groupe avec des prestataires de soins de santé issus de milieux de soins primaires en équipe en Ontario, au Canada. Les données ont été analysées à l’aide d’une analyse thématique.

**Résultats:** Nous avons organisé cinq groupes de discussion avec cinq équipes de soins primaires différentes, auxquels ont participé 31 prestataires de soins de santé. Nous avons construit quatre thémes (chacun avec des sous-thémes) liés aux expériences, aux obstacles et aux facilitateurs perçus, ainsi qu’aux recommandations relatives à la prestation de services primaires en équipe pour la prise en charge de la lombalgie chronique, y compris (1) les parcours de soins et les modéles de prestation de services, (2) les processus et l’organisation de l’équipe, (3) la culture et l’environnement de l’équipe, et (4) les besoins des patients et leur état de préparation.

**Conclusions:** Les équipes de soins primaires mettent en œuvre divers parcours de soins et modéles de prestation de services pour la prise en charge des patients atteints de lombalgie chronique, qui peuvent être influencés par des facteurs liés au patient, à l’équipe et à l’organisation. Les résultats ont des implications potentielles pour la recherche future et les innovations dans la pratique afin d’améliorer la façon dont les soins primaires en équipe sont dispensés aux patients atteints de lombalgie chronique.

## Introduction

Low back pain (LBP) is the leading contributor to years lived with disability worldwide^1,2^ and is one of the most common reasons patients seek primary care.^3–5^ LBP is also costly to the health care system. Patients with LBP incur health care costs at a rate approximately 60% higher than those without LBP.^6^

Chronic LBP (LBP that persists for greater than 3 months in duration^7^) is one of the most common chronic pain conditions.^8^ Evidence suggests that interprofessional management, including coordinated care provided by a team of health care providers, is superior to unimodal management for chronic pain conditions, such as chronic LBP.^9–12^ Despite this, in Canada, access to interprofessional chronic pain clinics is a challenge due to lengthy waitlists and geographic inequities.^13,14^ Furthermore, access to interprofessional chronic pain care has become increasingly more challenging as a result of impacts of the COVID-19 pandemic.^15^

Given issues with access to interprofessional chronic pain clinics,^13,14^ combined with calls to move away from biomedical and fragmented models of care,^16^ team-based models of primary care may offer an important care pathway for patients with LBP to access comprehensive interprofessional care. Across Canada, primary care is increasingly shifting to interprofessional team-based models of care.^17^ The Patient’s Medical Home is a vision for the future of primary care in Canada, which includes team-based care as a key foundation.^18^ Team-based primary care occurs when a patient receives care from the most appropriate member of an interprofessional primary care team and information is communicated efficiently among team members.^18^ Emerging evidence suggests that team-based approaches to primary care have benefits such as improving patient perceptions of care^19^ and reducing emergency department use.^20^ In Ontario, for example, team-based approaches to primary care exist^21^ and include models such as family health teams (FHTs)^22^ and community health centers (CHCs).^23^ These models of team-based primary care can include various health care providers, such as family physicians and/or nurse practitioners^24^ as well as pharmacists,^25^ dietitians,^26^ social workers,^27^ occupational therapists,^28^ and physiotherapists,^29^ among others.

Previous research has explored primary care physicians’ experiences with the primary care management of chronic pain in Canada,^30^ as well as perceived challenges of chronic pain management in community clinics in the United States.^31^ However, no research to date has specifically explored health care provider perspectives of the delivery of team-based primary care for the management of chronic LBP. Given potential aspects of care delivery that may be unique to chronic LBP in comparison to other chronic pain conditions, combined with an increasing shift toward team-based delivery of primary care across Canada,^17^ there is value in understanding the perspectives of health care providers from multiple professional backgrounds on this specific area of practice. A better understanding of primary care team perspectives will provide foundational knowledge on how primary care teams are providing care for patients with chronic LBP and has the potential to inform future research and practice innovations to improve how patients with chronic LBP are managed within this practice setting.

The purpose of this study was to understand health care providers’ experiences, perceived barriers and facilitators, and recommendations when providing team-based primary care for the management of chronic LBP.

## Materials and Methods

### Study Design

We conducted an interpretive description qualitative study.^32^ Interpretive description seeks to “provide a thematic or integrative description of a phenomenon of applied or practical interest.”^32(p38)^ We chose to approach this research using an interpretive description approach because it was aligned with our objectives to understand health care providers’ experiences, perceived barriers and facilitators, and recommendations when providing team-based primary care for the management of chronic LBP.

### Ethical Approval

Ethical approval for this was research was granted by the Queen’s University Health Sciences and Affiliated Teaching Hospitals Research Ethics Board in Kingston, Ontario, Canada (TRAQ No. 6029278).

### Theoretical Framework

We approached this research within a constructivist worldview, where we acknowledged that multiple realities can exist simultaneously.^33^ Practically, a constructivist epistemology influenced our selection of study methodology (e.g., selection of a qualitative methodology), approach to data collection (e.g., inviting and encouraging multiple perspectives through focus group discussions), data analysis (e.g., searching for diverse perspectives and multiple viewpoints by participants within focus groups and across focus groups), and presentation of results (e.g., selecting representative quotes that demonstrated diverse perspectives within a theme and/or subtheme). Although we approached data analysis inductively, our understanding of team-based primary care was broadly informed by concepts described in the Patient’s Medical Home model.^18^

### Research Team

Our research team included a physiotherapist and PhD candidate with clinical experience in interprofessional chronic pain care (K.V.), an occupational therapist and researcher with clinical experience providing team-based primary care (C.D.), two patient partners with lived experience of chronic LBP (G.N. and T.L.), a health psychology professor and researcher with expertise in pain (D.A.T.), and a physiotherapist and researcher with a research program focused on reducing pain-related disability (J.M.).

### Participants and Recruitment

Participants included any health care provider (e.g., family physician, occupational therapist, social worker, physiotherapist) who practiced within two team-based models of primary care in Ontario (i.e., FHTs and CHCs). We chose to recruit participants from these primary care models because FHTs and CHCs are the two most common models of team-based primary care in Ontario. In Ontario, over 3.4 million patients are rostered to a FHT,^22^ and there are 101 CHCs who provide care to priority populations.^23^ We used a purposive sampling technique^34^ with the goal of recruiting primary care teams who were from urban and rural contexts, different Ontario health regions, and different models of team-based primary care (i.e., FHTs and CHCs). We recruited primary care teams by contacting specific primary care team managers/executive directors and inviting health care providers on their teams to participate. We contacted eight primary care teams, of which five agreed to participate in the study. The three primary care teams that did not participate did not provide a reason for declining to participate. The Association of Family Health Teams of Ontario and Alliance for Healthier Communities assisted us by sending targeted emails to specific managers/executive directors.

### Data Collection

We developed a focus group discussion script based on study objectives (see Box 1).^35^ All focus groups were conducted virtually using Zoom, an online video conference software. The first author (K.V.) conducted all focus groups. All focus groups were recorded, transcribed verbatim, and reviewed for accuracy. The manager/executive director of each primary care team completed a brief questionnaire to better understand details on each primary care team. All participants provided verbal informed consent to participate in the research at the beginning of each focus group discussion. Focus groups were conducted from August to November 2020. When deciding to stop data collection, we drew upon concepts of information power.^36^ Information power allows researchers to determine an adequate sample size using a number of elements, such as the quality of dialogue with participants.^36^ Within the context of this research, we decided to stop data collection after conducting focus groups with five primary care teams based on the quality of dialogue with participants and specificity of the sample, which had in-depth knowledge and experience within team-based primary care settings.

**Box 1.** Sample questions from the focus group discussion script.

What has been your experience with team-based care for the management of chronic LBP in primary care? What are the benefits/drawbacks of providing team-based care for the management of chronic LBP in primary care? What patients with chronic LBP do you commonly provide team-based care for? What health care providers are involved in team-based care for the management of chronic LBP at your primary care site? What are the facilitators to providing team-based care for the management of chronic LBP at your primary care site? What are the barriers to providing team-based care for the management of chronic LBP at your primary care site? What are your recommendations to improve team-based care for the management of chronic LBP in primary care?

### Data Analysis

Interpretive description allows researchers to approach analysis from various interpretive techniques.^32^ We approached data analysis using thematic analysis as described by Braun and Clarke, including (1) familiarization with the data, (2) generating initial codes, (3) searching for themes, (4) reviewing themes, (5) defining and naming themes, and (6) producing a report.^37^ Consistent with an interpretive description approach, we continually asked ourselves questions such as “So what?” and “What does this mean clinically?” throughout the analysis process.^32^ As a first step, the first author (K.V.) reviewed each transcript to become familiar with the data by reading and re-reading the transcripts. To generate initial codes, the first and last author (J.M.) each independently coded one transcript before meeting to discuss initial codes, salient topics raised by participants, and overall impressions. Next, the first author independently coded the remaining transcripts using this initial coding structure, where new codes were continually added and existing codes were combined and/or refined as needed. Once all transcripts were coded, the first author searched for preliminary themes. Using a series of virtual meeting of members of the broader research team (C.D., T.L., G.N., D.T.), preliminary themes and subthemes were presented by the first author and reviewed by the entire team. Preliminary themes and subthemes were continually revised based on feedback from the research team through an iterative process. Next, we defined and named our themes and subthemes. As a final step, we selected representative quotations to illustrate our findings. We used MAXQDA (Version 11. Berlin: VERBI Software; 2014) to organize the data throughout our analysis.

### Rigor

We used multiple strategies to build trustworthiness and enhance the rigor of this work through use of an audit trail, analytic memos, and regular reflexive dialogue between diverse members of the research team (i.e., patient partners, health care providers, and researchers) throughout data analysis.^38,39^

## Results

We conducted five focus groups with five primary care teams, including a total of 31 health care providers. Each focus group was approximately 60 min in length. Four of the primary care teams were structured as FHTs, and one was a CHC. Four of the teams were situated within urban settings and one had both urban and rural locations. Teams were located in three of Ontario’s five health regions. Focus groups included diverse health care disciplines, including family physicians (*n* = 5), occupational therapists (*n* = 5), chiropractors (*n* = 4), pharmacists (*n* = 4), physiotherapists (*n* = 3), kinesiologists (*n* = 2), registered nurses (*n* = 2), dietitians (*n* = 2), a psychotherapist (*n* = 1), a social worker (*n* = 1), a nurse practitioner (*n* = 1), a system navigator (*n* = 1), and a health educator (*n* = 1). There was variation in the size of each focus group, with a range between three to eight participants. See Table 1 for characteristics of the primary care teams who participated in each focus group.

**Figure 1.**
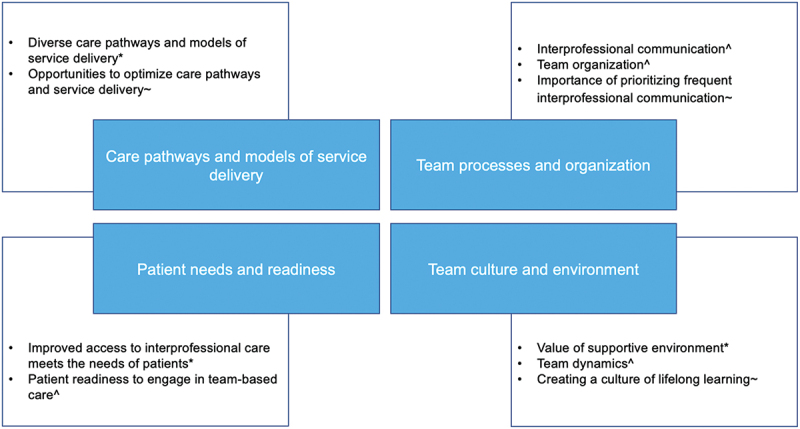
Themes and sub-themes related to healthcare providers experiences, perceived barriers and facilitators, and recommendations to providing team-based primary care for the management of chronic LBP.

*sub-theme describes an experience providing team-based primary care for the management of chronic LBP; ^sub-theme describes a perceived barrier and/or facilitator to providing team-based primary care for the management of chronic LBP; ~sub-theme describes a recommendation to improve the delivery of team-based primary care for the management of chronic LBP.

**Table 1.** Characteristics of primary care teams who participated in the focus groups.

	Focus group 1	Focus group 2	Focus group 3	Focus group 4	Focus group 5
Ontario health region	West	East	Central	East	West
Urban/rural practice site	Urban	Urban	Urban	Urban and rural	Urban
Type of team-based primary care practice	FHT	FHT	FHT	CHC	FHT

We constructed four themes (each with subthemes) related to health care providers’ experiences, perceived barriers and facilitators, and recommendations when providing team-based primary care for the management of chronic LBP, including (1) care pathways and models of service delivery, (2) team processes and organization, (3) team culture and environment, and (4) patient needs and readiness. See Figure 1 for a summary of themes and subthemes and their connections to study objectives.

### Theme 1: Care Pathways and Models of Service Delivery

Participants described varied experiences and recommendations related to care pathways and models of service delivery when providing team-based primary care for the management of chronic LBP.

#### Diverse Care Pathways and Models of Service Delivery

Participants described how they enact team-based care of patients with chronic LBP through a multipronged approach, including referrals between health care providers on the primary care team, structured interprofessional care pathways and/or programs, as well as referrals, when needed, to health care providers and services outside of primary care. For example, participants described how team-based care occurs via referrals between health care providers:

Usually [a patient with chronic low back pain] would be referred to me from […] the doc or the resident or medical team. […] But also I could get referred from the other allied health members. (System navigator, focus group 5)

Structured interprofessional care pathways, including a team of health care providers who specifically focus on musculoskeletal health, were one approach to providing team-based care for patients with chronic LBP:

So within [the musculoskeletal] program we have […] the chiropractor. We have […] the kinesiologists as well as […] the OT […] we triage the patient to determine which type of care they are best suited for. (Chiropractor, focus group 3)

Of note, participants described how many of their group-based programs had shifted to virtual and hybrid care delivery due to the COVID-19 pandemic, which had perceived benefits for patients with chronic LBP:

We’ve gone remotely [in our chronic pain management program], and virtual [care] has actually been a way that we’ve actually been able to see more people [… it] is our highest attendance group so far […] the no-show rate is just super low. (Physiotherapist, focus group 1)

Depending on the complement of health care providers on the primary care team, participants also described how they provide team-based care by referring patients to health care providers and services external to the primary care team when needed:

Occasionally I will make community referrals, like to different physiotherapists throughout the Arthritis Society or through home and community care. (Occupational therapist, focus group 2)

#### Opportunities to Optimize Care Pathways and Service Delivery

Participants described various recommendations to optimize care pathways and models of service delivery when providing team-based primary care for the management of chronic LBP. Participants recommended creating more direct patient care pathways, increasing the delivery of group-based programs, as well as creating more collaborative care models with health care providers and services outside of primary care. For primary care teams where policies dictated that patients could not directly access certain health care providers without a referral from a family physician or nurse practitioner, participants recommended creating care pathways that allow for more direct patient access:

My only wish is that some of these patients could have quicker access to my services so I could see them quicker. (Physiotherapist, focus group 5)

Participants acknowledged how traditional care pathways and internal policies that involve or require a family physician or nurse practitioner to act as the first point of contact and who then refers to other health care providers on the primary care team can create bottlenecks in care for patients:

I think ideally it would be great to have […] open access [for patients] to sort of any member of the team. […] I find part of what bottlenecks some patients is that they need to see a physician as a gatekeeper when often times they […] don’t necessarily need my services. They just need me to get to the next step, the person they actually want to see. (Family physician, focus group 2)

Participants also recommended the value of creating more group-based care options for patients with chronic LBP, where patients can interact with and learn from their peers:

I think [patients] need more program-based [care options] where they can go through this a bit more with other people in a program setting […] because sometimes me [as a health care provider] saying it is not going to be the thing. They need to be around others that are facing these issues. (Occupational therapist, focus group 1)

Finally, participants who were part of teams that did not have certain health care disciplines recommended creating more collaborative care models with health care providers and services outside of the primary care setting to strengthen the provision of team-based primary care. For example, one participant highlighted the value of creating more collaborative care pathways with specialty chronic pain clinics when providing care for patients with chronic LBP:

I would love to increase our collaboration with the specialty pain clinics and basically work with them more closely, especially for shared patients. […] So how can we bring some of that care closer to home but still maintain the specialty kind of lens. (Pharmacist, focus group 2)

### Theme 2: Team Processes and Organization

Participants described perceived barriers and facilitators and recommendations related to team processes and organization when providing team-based primary care for the management of chronic LBP.

#### Interprofessional Communication

Participants described how interprofessional communication mechanisms are an important team process that can serve as a facilitator to providing team-based primary care for the management of chronic LBP. Participants described how a shared electronic medical record facilitates the delivery of team-based care for patients with chronic LBP:

[A] perk that comes with being in the same building is often to be on the same EMR […] we have a great EMR […] it’s just kind of like you can send a quick message from doctor to [interprofessional health care provider], so it’s great […] there’s [nothing] lost [in] communication. (Chiropractor, focus group 3)

Regular in-person communication among members of the primary care team was another communication mechanism described as a facilitator to providing team-based care for patients with chronic LBP:

The best facilitator is that we are in such close proximity to each other and, you know, there’s lots of hallway consults and lots of regular communication. (Family physician, focus group 5)

#### Team Organization

Participants described how team organization, including team complement and co-location, served as either a barrier or facilitator to providing team-based primary care for the management of chronic LBP. For some primary care teams, participants described how not having certain health care disciplines on the primary care term served as a barrier to providing team-based care for patients with chronic LBP:

I would agree that […] not having a physiotherapist or a chiropractor or someone else who can really focus on the mechanical nature of pain is a challenge. (Occupational therapist, focus group 2)

Participants also described how the physical organization of health care providers on the primary care team impacted the delivery of team-based care. For example, participants described how co-location of health care providers was a facilitator to the delivery of team-based primary care:

I have been part of family health teams in the past that logistically were not set up this way [co-located. …] They would have an allied health building and then we would be in a separate building from the physicians. And it really wasn’t that same level of collaboration that there is with us being in one location. (Pharmacist, focus group 5)

Conversely, participants described that when health care providers on a primary care team are not always co-located at one physical location, this can serve as barrier to the delivery of team-based care for patients with chronic LBP:

[W]e’re not always all at the same site or […] location. […] And sometimes you’d like to consult the physician or the nurse practitioner or the nurse or the dietitian, but they might not be in the office right now. […] So then you kind of postpone it and then, we’re all human, and we don’t necessarily touch back on that on that patient. (Chiropractor, focus group 4)

#### Importance of Prioritizing Frequent Interprofessional Communication

Participants provided recommendations related to team processes, including the importance of prioritizing frequent interprofessional communication when providing team-based primary care for patients with chronic LBP. For example, participants recommended implementing regular interprofessional team rounds:

In my perfect world it would be nice to either have […] more frequent kind of interprofessional rounding with respect to pain patients because we know they can, without I guess proper monitoring, things can kind of slip through the cracks. (Physiotherapist, focus group 1)

Other participants recommended increasing the frequency of communication between family physicians and other health care providers on the primary care team:

[M]aybe just getting back with the physician a bit more frequently in a more formal way. So rather than just relying on EMR updates maybe more conversations, whether that be in person or via a virtual means. (Occupational therapist, focus group 2)

Other participants provided recommendations to have more frequent communication between patients and relevant health care providers on the primary care team:

I don’t think it’s been done yet, but if need be, having a three-way meeting, right, with the nutritionist, the nurse practitioner, or the physician as well as the chiropractor to be like, look this is our global idea. This is what we want to do with you [the patient. …] We do have a good communication basis, but I think it could be improved. (Chiropractor, focus group 4)

### Theme 3: Team Culture and Environment

Participants described overall experiences, perceived barriers and facilitators, and recommendations related to team culture and environment when providing team-based primary care for the management of chronic LBP.

#### Value of Supportive Environment

Participants described overall positive experiences providing team-based primary care for the management of chronic LBP because of the supportive environment between health care providers on the primary care team. For example, participants valued having support from the rest of the primary care team when providing care for patients with chronic LBP, given that some patients have a complex presentation:

I find it much more supportive having a team, especially within my profession. So other physios as well as outside my profession to help with patients that are dealing with chronic [low back] pain. It’s often a bit of a complex presentation where it’s nice to have more than one lens and well as more than one head coming together, trying to offer a range of skills. (Physiotherapist, focus group 1)

Participants further described how a supportive environment improves their experience when providing care for patients with chronic LBP:

Certainly, from […] my own perspective, the support […] is amazing. You know, sometimes you just don’t know what to do with somebody. […] I can’t imagine practicing without the team actually […] because I think it can be an extremely overwhelming group of people to work with at times. (Occupational therapist, focus group 5)

#### Team Dynamics

Participants described how team culture and environment, including dynamics among members of the primary care team, can serve as a barrier or facilitator when providing team-based primary care for patients with chronic LBP. For example, one participant described how challenging dynamics between members of the primary care team served as a barrier to providing team-based care:

Sometimes I think we […] all struggle with the primary care physician allowing us to sort of do what we think is really beneficial. […] I think that that hopefully in the next year or two the value of how we collaborate will be appreciated by everyone. (Psychotherapist, focus group 3)

In another team, a team dynamic where collaboration was respected and there was no perceived hierarchy among members of the team was perceived as a facilitator to providing team-based care for patients:

I think the thing that makes us work really, really well together is that there is no hierarchy here. We are a team. Like, it’s not just in the name, it truly is, we are a team. I don’t think I’m any better or less than the physician. I feel like I have a strong knowledge base and I have information to share and I feel valued. I feel like my assessments are valued and that my opinions are valued. (System navigator, focus group 5)

#### Creating a Culture of Lifelong Learning

Participants provided recommendations related to team culture and environment, including the value of creating a culture of lifelong learning. Given that practice approaches for the management of chronic pain conditions such as chronic LBP have changed over time, participants described the importance of continual life learning:

I went through my pharmacy education at a time where we were just starting to talk about deprescribing opioids and the risks associated with opioids. And the pendulum has really shifted dramatically from […] the time that I was a learner to being a practicing clinician. So I think a lot of that learning had to be done on the job because the entire environment has changed. […] So all of that for me has been learned through [continuing education]. (Pharmacist, focus group 2)

Participants recommended creating a culture where health care providers on the primary care team are willing to learn from each other’s disciplinary knowledge:

I think […] willingness to learn is super important. And acknowledging [as a health care provider] you might be really good at what you do but knowing that you’re not an expert in everything and really asking each other questions and pulling on all of the great resources that we have here [as a team]. (Chiropractor, focus group 3)

Ultimately, participants recommended creating a culture where members of the primary care team regularly participate in professional development to enhance knowledge and competence when caring for patients with chronic LBP:

[T]he ECHO Pain Program […] I just think that it’s so helpful because you have so many different specialists at the table. […] So it’s such a learning opportunity […] I’m really trying to encourage more people [on the team] to be taking advantage of those things. (Family physician, focus group 1)

### Theme 4: Patient Needs and Readiness

Participants described overall experiences and perceived barriers and facilitators related to patient needs and readiness when providing team-based primary care for the management of chronic LBP.

#### Improved Access to Interprofessional Care Meets the Needs of Patients

Participants described overall experiences of how team-based primary care helps to meet the needs of patients with chronic LBP by improving access to interprofessional health care providers and programs. Participants acknowledged how team-based care is ideal for patients with chronic LBP; however, it is often not accessible to those who need it most. For example, participants described how team-based primary care helps to improve patient access to the care they need:

[W]e’re […] giving people access to care that would likely […] be unable to afford [through] private care. […] We […] take a team-based approach and help our patients get the care that they need. (Kinesiologist, focus group 3)

Participants further described how team-based primary care meets the needs of patients with chronic LBP because many patients would not otherwise be able to access care from an interprofessional team:

I don’t want to say a lot of our clients, but enough of our clients are less fortunate. So it’s nice that we’re able to offer that opportunity [to access interprofessional care] for them because a lot of them can’t afford it. (Registered nurse, focus group 4)

Ultimately, participants described how they valued providing team-based primary care for patients with chronic LBP because it improved access to needed services:

I’m very thankful that I work with a family health team where we have allied health available for patients and in order to provide a lot of services that I think we wouldn’t be able to otherwise, especially for people who don’t have private coverage. And, for example, OT [occupational therapy] and the systems navigator I think would be very hard to access even with people who have private coverage. (Family physician, focus group 5)

#### Patient Readiness to Engage in team-based Care

Although participants reported that team-based models of primary care improve access to interprofessional health care providers and programs for patients with chronic LBP, they also described how patient readiness to engage in team-based care can serve as either a barrier or facilitator. Participants described how they perceived that some patients with chronic LBP are not ready to engage in more active management strategies that are commonly provided by interprofessional health care providers. For example, participants described how some patients are not ready to engage in team-based care:

[U]nfortunately, the level of readiness and the right kind of motivation unfortunately is not always there. (Occupational therapist, focus group 1)

Conversely, other participants highlighted how some patients with chronic LBP are eager and ready to engage in care delivered by interprofessional health care providers, which served as a facilitator to the delivery of team-based primary care:

[W]e have a smaller subgroup of people who are just like ready to learn anything. They’re super grateful with everything we tell them. (Kinesiologist, focus group 3)

Although readiness to engage in team-based care was described as a barrier when providing team-based primary care for some patients with chronic LBP, participants acknowledged the important interplay between readiness to engage in care within the context of a patient’s life circumstances, particularly within the context of chronic pain:

People with more chaotic, demanding lives aren’t as able to, you know, work in a management plan that is suggested to them. […] It’s not just about [readiness] stages of change and whether they’re there yet or not. It’s all of those different layers. (Family physician, focus group 5)

## Discussion

To our knowledge, this is the first study to explore health care providers’ experiences, perceived barriers and facilitators, and recommendations when providing team-based primary care for the management of chronic LBP. We constructed four themes (each with subthemes): (1) care pathways and models of service delivery for chronic pain, (2) team processes and organization, (3) team culture and environment, and (4) patient needs and readiness. Overall, our results suggest that team-based primary care contributes to overall positive experiences for health care providers when providing care for chronic LBP. Our results contribute to an improved understanding of team-based primary care for the management of chronic LBP and have implications for future research and practice innovations. Although our results are specific to team-based primary care of patients with chronic LBP, aspects of the results may be transferable to other chronic pain conditions and health system contexts.

Webster et al. previously explored experiences with chronic pain management in the context of primary care in Canada and found that primary care physicians experience challenges providing care for patients with chronic pain, particularly those who struggle with poverty, addiction, and other mental health issues.^30^ Previous work by Upshur and colleagues also explored primary care provider concerns with chronic pain management in the United States and found that providers were dissatisfied with their training on chronic pain management and that they faced struggles related to patient self-management and opioid prescribing.^31^ Our results complement this previous work by specifically exploring perspectives of health care providers from multiple professional backgrounds of team-based primary care for the management of chronic LBP.

Participants described experiences with diverse care pathways when providing team-based primary care for the management of chronic LBP, as well as recommendations to optimize approaches to service delivery. This finding aligns with recent calls to action to improve pathways and models of care for patients with LBP and other chronic pain conditions.^16,40^ Given that this work was conducted during the COVID-19 pandemic, participants described how some of their services for patients with chronic LBP had adapted to virtual and hybrid models, which is consistent with previous findings on interprofessional primary care practice during the global pandemic.^41^ Participants in this research described specific recommendations, such as allowing patients with chronic LBP to directly access any member of the primary care team without a referral by a family physician or nurse practitioner. This is consistent with concepts of team-based care described in the Patient’s Medical Home, where a patient does not always see their family physician but has interactions with all members of the primary care team.^18^ Research efforts are currently underway to explore care pathways, such as models that encourage patients with LBP or other musculoskeletal conditions to see physiotherapists as their first contact within the primary care team in both Canada and England.^42–44^ These care pathways have the potential to be an effective approach that could be scaled up and implemented within team-based models of primary care to optimize service delivery, decrease use of unnecessary health resources, and improve patient outcomes. Furthermore, given recommendations by our participants to prioritize the delivery of group-based programs and collaborative models of care with services outside of primary care, this may provide an important direction for future research and practice innovations.

Our results demonstrate the importance of team processes and organization when providing team-based care for patients with chronic LBP. Participants in this research described how interprofessional communication mechanisms, such as a shared electronic medical record, are an important facilitator to the delivery of team-based care. This finding is consistent with concepts described in the Patient’s Medical Home, whereby appropriate infrastructure, including a shared electronic medical record, is a foundation of quality primary care delivery.^18^ Our participants also described how team organization, such as the physical organization of the primary care team, served as either a barrier or facilitator to the delivery of team-based primary care for patients with chronic LBP. These findings are consistent with previous research that found that co-location of health care providers in primary care can improve care integration.^45^ Although primary care teams do not have to be co-located to provide quality care, they do need to have clear communication channels between health care providers on the team.^18^ Given that our participants recommended the importance of prioritizing interprofessional communication, primary care teams should not underestimate the importance of prioritizing frequent collaborative communication between health care providers when providing care for patients with chronic LBP.

Participants also described overall experiences, perceived barriers and facilitators, and recommendations related to team culture and environment when providing team-based primary care for the management of chronic LBP. Of note, participants described how they valued the supportive environment created when providing care alongside a team of health care providers. Given health care providers’ perceived challenges in providing primary care for patients with chronic pain conditions such as chronic LBP,^30,31^ our findings suggest that team-based approaches to primary care may help to enhance health care providers’ experiences with care delivery for this patient population, which is an important aspect of the quadruple aim.^46^ In alignment with existing interprofessional collaboration frameworks,^47^ participants in our research described the important impact of team dynamics when providing team-based primary care. Therefore, primary care teams should consider strategies to enhance and support team functioning, with the goal of improving both health care provider experiences and patient outcomes. Participants also recommended the importance of creating a team culture of lifelong learning among the primary care team. Given limitations with health care provider prelicensure education on pain,^48^ health care providers in primary care teams should prioritize engagement in continuing professional development specific to chronic pain management. In Ontario, for example, professional development opportunities exist for primary health care providers specific to chronic pain management, such as Project ECHO, which have shown positive results.^49,50^

Finally, participants in our research described overall experiences and recommendations related to patient needs and readiness when providing team-based primary care for the management of chronic LBP. Participants described how team-based models of primary care help to meet the needs of patients by improving access to interprofessional health care providers and services such as social workers, physiotherapists, and pharmacists. Given increasing challenges with access to interprofessional chronic pain clinics in Canada,^13,14^ which has been compounded by the COVID-19 pandemic,^15^ our findings highlight how team-based approaches to primary care may offer an alternative option for patients with chronic low back pain to receive comprehensive and interprofessional care. This notion is supported by evidence that collaborative models of care for the management of chronic pain can lead to improved patient outcomes in the primary care setting.^51^ However, given that primary care teams provide care for a broad range of health issues,^52^ it may be important to also consider strategies to ensure that primary care teams have appropriate and up-to-date skills and confidence to effectively manage patients with chronic pain conditions such as chronic LBP. Despite the perceived value of team-based primary care for patients with chronic LBP, participants also described barriers related to patient readiness to engage in team-based care. Readiness to engage in active and self-managed chronic pain management strategies has been broadly explored in literature.^53–55^ This has important practical implications and highlights the potential role for health care providers to use patient-reported outcome measures, such as the Pain Stages of Change Questionnaire,^56^ to evaluate patient readiness to engage in team-based care for the management of chronic LBP.

### Limitations

This research is not without limitations. First, all participants were from primary care teams in Ontario, Canada. Therefore, it is unclear how transferable the results are to other regions across Canada and internationally. Second, none of our participants were from primary care teams in remote or northern Ontario regions. Given unique contextual factors with health care delivery in these areas,^57^ future work may benefit from specifically exploring the delivery of team-based primary care for the management of chronic LBP in this context. We focused this research on the two most common models of team-based primary care in Ontario (i.e., FHTs and CHCs) but did not include participants from other models of team-based primary care that also exist (i.e., Aboriginal health access centers^58^ or nurse practitioner–led clinics^59^). Therefore, our results may not be transferable to these other models of primary care, and this may be a valuable area of exploration for future research. It is also important to acknowledge that the primary care teams who agreed to participate in this research may have perceived that they were more positively delivering team-based primary care for patients with chronic LBP than the average primary care site. As such, it is possible that the results included within this study represent a more positive perspective than would have been gleaned from other primary care teams. Finally, although we asked participants about their experiences, perceived barriers and facilitators, and recommendations when providing team-based primary care for the management of chronic LBP, it is possible that participants may have drawn from their experiences in providing care for patients with chronic pain broadly. Therefore, some of the participant responses may represent perspectives of team-based primary care for the management of chronic pain conditions broadly versus being specific to chronic LBP.

## Conclusions

We constructed four themes (each with subthemes) related to health care providers’ experiences, perceived barriers and facilitators, and recommendations when providing team-based primary care for the management of chronic LBP, including (1) care pathways and models of service delivery, (2) team processes and organization, (3) team culture and environment, and (4) patient needs and readiness. Overall, health care providers described positive experiences delivering team-based primary care for the management of chronic LBP. We found that primary care teams are implementing diverse care pathways and models of service delivery for the management of patients with chronic LBP, which can be influenced by patient, team, and organizational factors. Recommendations when providing team-based primary care centered around the importance of optimizing care pathways and service delivery, prioritizing interprofessional communication, and a commitment to lifelong learning and professional development. Results contribute to an understanding of the current landscape of team-based primary care for the management of chronic LBP from the perspective of health care providers and can serve as a point of departure for future research and practice innovations. The results may be transferable to other chronic pain conditions and health system contexts.
